# Cytokine and immune cell profiling in the cerebrospinal fluid of patients with neuro-inflammatory diseases

**DOI:** 10.1186/s12974-019-1601-6

**Published:** 2019-11-14

**Authors:** Gildas Lepennetier, Zsuzsanna Hracsko, Marina Unger, Martijn Van Griensven, Verena Grummel, Markus Krumbholz, Achim Berthele, Bernhard Hemmer, Markus C. Kowarik

**Affiliations:** 1 Department of Neurology, Klinikum rechts der Isar, Technical University of Munich, Munich, Germany; 2 Department of Internal Medicine 1, Universitätsklinikum Erlangen, Friedrich-Alexander Universität Erlangen-Nürnberg, Erlangen, Germany; 3 Department of Experimental Trauma Surgery, Klinikum rechts der Isar, Technical University of Munich, Munich, Germany; 4 Department of Neurology and Hertie Institute for Clinical Brain Research, Eberhard Karl University, Tübingen, Germany; 5 Munich Cluster for Systems Neurology (SyNergy), Munich, Germany

**Keywords:** Cytokines, Neuro-inflammation, Immune cell subsets, CSF, B cells, NK cells, CXCL13, Blood-brain barrier

## Abstract

**Background:**

Cytokines play multiple roles during neuro-inflammatory processes and several cytokines have been studied in the context of specific diseases. This study provides a comprehensive picture of cerebrospinal fluid (CSF) changes during neuro-inflammation by analyzing multiple cytokines in combination with immune cell subsets and standard CSF parameters.

**Methods:**

Using multiplex assays, we simultaneously measured 36 cytokines (CCL1–3, CCL7, CCL8, CCL11, CCL13, CCL19, CCL20, CCL22–27, CXCL1, CXCL2, CXCL5, CXCL6, CXCL8, CXCL9, CXCL11–13, CXCL16, CX3CL1, IL2, IL4, IL6, IL10, IL16, GM-CSF, IFNγ, MIF, TNFα, and MIB1β) in the CSF and serum of 75 subjects. Diagnoses included clinically isolated syndrome and relapsing-remitting multiple sclerosis (MS, *n* = 18), secondary progressive MS (*n* = 8), neuro-syphilis (*n* = 6), Lyme neuro-borreliosis (*n* = 13), bacterial and viral meningitis (*n* = 20), and patients with non-inflammatory neurological diseases (NIND, *n* = 10). Cytokine concentrations were correlated with CSF standard parameters and CSF immune cell subsets (CD4 and CD8 T cells, B cells, plasmablasts, monocytes, and NK cells) quantified by flow cytometry.

**Results:**

We observed increased levels of multiple cytokines (26/36) in patients with neuro-inflammatory diseases when compared to NIND that consistently correlated with CSF cell count and Q_Albumin_. Most CSF cytokine concentrations correlated with each other, but correlations between CSF and serum values were scarce (3/36). Within the CSF compartment, CXCL13 showed a strong association with B cells when analyzing all patients, as well as patients with an intact blood-brain barrier (BBB). NK cells positively correlated with CSF concentrations of multiple cytokines (22/36) when analyzing all patients. These correlations were maintained when looking at patients with a disrupted BBB but not detectable in patients with an intact BBB.

**Conclusions:**

Under conditions of neuro-inflammation, multiple CSF cytokines are regulated in parallel and most likely produced locally. A combined increase of CSF CXCL13 levels and B cells occurs under conditions of an intact BBB. Under conditions of a disrupted BBB, CSF NK cells show significantly increased values and seem to have a major contribution to overall inflammatory processes, reflected by a strong correlation with multiple cytokines. Future studies are necessary to address the exact kinetics of these cytokines during neuro-inflammation and their relation to specific diseases phenotypes.

## Background

Cytokines are small proteins that are secreted by various cell types and play multiple roles during development, homeostasis, and immune regulation [6]. According to their functions, cytokines are classified into tumor necrosis factors, adipokines, interleukins (communication between leukocytes), interferons, and chemokines [10, 26]. During inflammation, cytokines serve as chemotactic factors, moderate cell-to-cell communication, and regulate immune cell differentiation [4]. Regarding neuro-inflammation, several cerebrospinal fluid (CSF) cytokines have been implicated in autoimmune as well as infectious diseases. However, investigations that focused on CSF cytokines in patients with neurological diseases have mainly been restricted to specific diseases.

Several studies on CSF cytokines are available in patients with multiple sclerosis (MS). CSF levels of TNF-α, IL12, CXCL9, CXCL10, CCL17, CCL21, CCL22, IL10, IL6, IL23, IL17, IL8, CXCL13, CCL19, and CCL5 (also called RANTES in references) have been shown to be consistently upregulated [19, 21–23]. Increased levels of CXCL10 (IP-10), CXCL9 (MIG), and CCL5 were reported in MS patients with acute relapse [31, 41]. Latest studies showed elevated CSF values for IL27 as well as IL2RA, CCL5, CCL11, MIF, CXCL1, CXCL10, SCF, and TRAIL [18, 27] whereas inconsistent results are available for CCL2, CCL3, and CCL4 [19]. In the spirochetal CNS infections Lyme neuro-borreliosis (LNB) and neuro-syphilis (Lues), distinct changes in the CSF cytokine profile have been found. CXCL13 has been shown to be significantly elevated in the CSF during active infection in both spirochete diseases [3, 9, 38, 40]. However, increased CXCL13 values are not specific for spirochete infections but are associated with a strong B cell recruitment in the CSF [20]. In addition, CSF concentrations of CXCL10, CCL2 (MCP-1), CCL3, CCL4, CCL5, IL8, and CXCL12 (SDF-1α) have been shown to be elevated in LNB [15, 35]. Urokinase plasminogen activator (uPA) and CXCL2 CSF values were elevated in patients with neuro-syphilis [29, 43]. In bacterial meningitis, multiple cytokines including CXCL10 (IP10), CCL2, CCL7 (MCP-3), CCL4 (MIP-1β), CCL5, CXCL12, IL6, IL8, and IL17 have been shown to be increased in the acute phase of the disease ([35]; Pinto [25, 36]). Elevated CXCL5, CXCL8, and CXCL1 and TNFα CSF concentrations have been reported in children with bacterial meningitis [32, 44]. Further attempts aimed to distinguish between different pathogens based on certain cytokine patterns. Hereby, TNFα and IFNγ showed higher values in pneumococcal than in meningococcal meningitis [7] and an upregulation of IL1β was suggested to discriminate between bacterial and aseptic meningitis [34]. Concerning viral infections, several studies detected elevated CSF levels of IL6, IL8, IL10, IL12, CXCL9, CXCL10, CXCL11, CCL2, CCL5, IL1β, TNF-α, BAFF, APRIL, IFN-α, and IFNγ in patients with viral meningoencephalitis with partially inconsistent results [19]. However, reliable clinical cytokine tests to discriminate between bacterial and viral CNS infections are not available yet.

In order to obtain a more comprehensive picture of CSF cytokine profiles in neurological diseases, we measured the concentrations of 36 cytokines in the CSF and serum of 75 patients using multiplex assays. Diagnoses included non-inflammatory controls (NIND), multiple sclerosis (RRMS) and clinically isolated syndrome (CIS), secondary progressive MS (SPMS), neuro-syphilis (Lues), Lyme neuro-borreliosis (LNB), and bacterial and viral meningitis. Cytokine values were correlated with CSF standard parameters such as CSF cell count, albumin quotient (Q_Albumin_), and immunoglobulin (Ig) indices. In addition, we also performed correlation analyses with different CSF immune cell subsets including CD4 and CD8 T cells, B cells, plasmablasts, NK cells, and monocytes that have been routinely quantified by flow cytometric analyses.

## Methods

### Patient characteristics

All patients were recruited at the Department of Neurology of the Technical University of Munich. CSF samples were obtained for routine diagnostic work-up, and patients consented to the scientific use of their biosamples. The ethics committee of the Technische Universität München approved the scientific use of CSF biosamples. Patients with non-inflammatory diseases (NIND, *n* = 10), relapsing-remitting multiple sclerosis (RRMS, *n* = 10), clinically isolated syndrome (CIS, *n* = 8), secondary progressive MS (SPMS, *n* = 8), neuro-syphilis (Lues, *n* = 6), Lyme neuro-borreliosis (*n* = 13), and bacterial (*n* = 10) and viral meningitis (*n* = 10) were included in our analysis. Patients with NIND suffered from normal pressure hydrocephalus (*n* = 3) and pseudotumor cerebri (*n* = 7). In patients with bacterial meningitis (*n* = 10), the following specific pathogens were identified: *Streptococcus pneumonia* (*n* = 2), *Haemophilus influencae* (*n* = 2), *Listeria monocytogenes* (*n* = 1), suspicion of *Mycobacterium tuberculosis* (*n* = 3), and undetermined, presumably bacterial pathogens (*n* = 2). In viral meningoencephalitis (*n* = 10), varicella zoster virus (*n* = 2), herpes simplex virus (*n* = 3) and undetermined presumably viral pathogen (*n* = 5) were detected. Further details are displayed in Table 1. Since all patients with CIS and RRMS were analyzed during relapse (clinical relapse or new MRI lesions), we merged these two patient groups for further analyses (CIS-RRMS).

**Table 1:** Basic patient characteristics. For each disease group, summary statistics are shown. Values are presented as average (minimum/maximum); for gender, numbers for female/male patients are displayed

Disease (*n*)	Gender	Age (years)	Preparation (minutes)	Disease duration (days)	Applied treatments (*n*)
NIND (10)	8/2	43 (22/85)	35 (20/50)	15.8 (0/74)	Acetazolamide (1), topiramate (1), no therapy (8)
CIS-RRMS (18)	16/2	35 (20/52)	46 (28/100)	19.4 (0/133)	Interferon beta (1), no therapy (17)
SPMS (8)	4/4	54 (40/68)	42 (25/60)	202 (62/343)	Mitoxantrone (3), rituximab (1), no therapy (4)
Lues (6)	0/6	41 (26/52)	48 (8/110)	0.9 (0/1.5)	Penicillin (3), ceftriaxone (1), no therapy (2)
LNB (13)	1/12	54 (19/80)	49 (30/85)	4.2 (0/31)	No therapy (3), all other patient treated with drugs including ceftriaxone, ampicillin, meropenem, amnd doxycycline
Bacterial meningistis (10)	5/5	59 (24/88)	49 (35/65)	7.2 (0.5/40.5)	All patient treated with drugs including ampicillin, gentamicin, ceftriaxone, vancomycin, penicillin, meropenem, isoniazid, rifampicin, streptomycin, cotrimoxazol, moxifloxacin, amoxicillin, and clavulanic acid
Viral meningitis (10)	5/5	52 (34/73)	47 (30/60)	6.7 (0/24)	1 patient without information, all other treated with drugs including aciclovir, ampicillin, and ceftrioxan

### Specimen handling and routine CSF testing

During the routine diagnostic work-up, 5 to 15 mL of CSF was obtained by lumbar spinal tap with an atraumatic needle. At the same occasion, 10 mL of EDTA blood was drawn for immunophenotyping and 10 mL of whole blood for serum analysis of albumin and immunoglobulins. Samples were processed according to the BioMS guidelines [42] and stored at − 80 °C for future cytokine measurements. The average preparation time between sample collection and freezing was 45 min.

For routine CSF work-up, CSF mononuclear cells were immediately counted in a Fuchs-Rosenthal chamber (Roth, Karlsruhe, Germany) to obtain CSF cell count. Total protein, albumin, IgG, IgM, and IgA concentrations in CSF and serum were determined by nephelometry according to the manufacturer’s instructions (Siemens ProSpec®, Eschborn, Germany). Oligoclonal bands were investigated by isoelectric focusing followed by silver staining.

### Immunophenotyping

Flow cytometric analysis of immune cell subsets was performed as described previously [20]. Shortly, fresh CSF was immediately spun down (300 g for 10 min), the supernatant removed, and the pellet resuspended in phosphate-buffered saline (PBS) (PAA, Pasching, Austria) with 2% fetal calf serum (FCS) (Invitrogen, Darmstadt, Germany). After incubating with our antibody mix (20 min at 4 °C), cells were spun down, washed, and resuspended in PBS wash solution (including 2% FCS) for flow cytometric analysis (Beckman Coulter Cyan, Brea, CA, USA). The following antibodies were used for staining: CD4 PerCP, CD3 APC-Cy7, CD45 VM (all BD Bioscience, Bedford, MA, USA), CD19 ECD, CD56 APC, CD14 FITC, and CD138 PE (all Beckman Coulter). This allowed differentiating CD4 T cells (CD45^+^CD3^+^CD4^+^), CD8 T cells (CD45^+^CD3^+^CD8^+^), monocytes (CD45^+^CD14^+^), NK cells (CD45^+^CD56^+^), B cells (CD45^+^CD19^+^CD138^−^), and plasmablasts (CD45 CD19^+^CD138^+^).

### Multiplex assays

In order to measure several cytokines in parallel, we performed multiplex assays (BioRad #171304070 M (10-Plex), #171AK99MR2 (40-Plex)) according to the manufacturers’ instructions. The 40-Plex kit contained the following cytokines: CCL1, CCL11, CCL13, CCL15, CCL17, CCL19, CCL2, CCL20, CCL21, CCL22, CCL23, CCL24, CCL25, CCL26, CCL27, CCL3, CCL7, CCL8, CX3CL1, CXCL1, CXCL10, CXCL11, CXCL12, CXCL13, CXCL16, CXCL2, CXCL5, CXCL6, CXCL8, CXCL9, GM-CSF, IFNγ, IL10, IL16, IL1β, IL2, IL4, IL6, MIF, and TNF-α. The 10-Plex Kit included the cytokine antibodies GCSF, IL12, IL17A, IL2, IL4, IL5, IFNγ, MIB1β, CCL5 (RANTES), and TNF-α. In order to enable an optimal comparability between different patient groups within one compartment, all CSF samples were measured on a single multiplex plate; all serum samples were measured on a single multiplex plate for each kit, respectively. CSF samples were applied undiluted; serum was pre-diluted 1:4. Multiplex plates were measured on a Luminex MAGPIX®. Standard curves and values were calculated using xPONENT 4.2 software for MAGPIX®. The required amount of 50 beads per analyte was consistently detectable for all analytes in the 40-Plex Kit. Although the 10-Plex Kit was used according to the manufacturer’s instructions, less than the required 50 beads per analyte were detectable for most cytokines. For this reason, only MIB1β from the 10-Plex Kit (> 50 beads in all samples) was used for further analyses in order to ensure good data quality. Standard curve ranges with upper and lower limits of cytokine concentrations are displayed in Additional file 6: Table S1. Cytokines with less than five observations in the control group (NIND) were completely excluded (in CSF: CCL17, IL1β; in serum: CCL21, IL1β). Additionally, when less than half of the measurements were in range, the cytokine was also excluded (CSF: CXCL10; serum: CCL15, CCL17). If the comparison between CSF and serum was not possible, the cytokine was finally removed for subsequent analyses (CCL15, CCL17, CCL21, CXCL10, and IL1β).

### Statistical analysis

All analyses and figures were done in R (version 3.5.3), using packages ggplot2 (3.3.1) and dplyr (0.8.1). The non-parametric Mann-Whitney rank sum test was used to compare cytokine concentrations in different conditions. The Shapiro-Wilk normality test was used to test the normal distribution of the data. In case of normality, Pearson’s correlation test was used; otherwise, Spearman’s non-parametric correlation test was used to test for correlation between cytokine concentrations. *p* values below 0.05 were considered significant. Bonferroni’s correction (correction for multiple testing of cytokine/immune cell subsets) was systematically used during statistical testing to reduce false positives.

## Results

### CSF standard parameters and immune cell distribution

CSF standard parameters such as cell count, glucose, lactate, albumin quotient (Q_Albumin_), IgG, IgA, and IgM index showed disease-specific changes within expected limits (Additional file 7: Table S2).

Regarding absolute numbers of CSF immune cell subtypes, B cells, CD4, and CD8 T cells were significantly elevated in LNB, Lues, bacterial meningitis, viral meningitis, and CIS-RRMS when compared to our control group with NIND. Plasmablasts only showed significantly increased values in patients with CIS-RRMS. Elevated NK cell numbers were observed in CIS-RRMS and bacterial and viral meningitis (Table 2 and Table 3).

**Table 2:** Percentage distribution of CSF immune cell subtypes in different neurological diseases. Values are given as mean ± standard deviation

Disease	CD4 T cells %	CD8 T cells %	Monocytes %	B cells %	Plasmablasts %	NK cells %
NIND	71 ± 12	16 ± 9	5 ± 4	0.4 ± 0.5	0 ± 0	2.7 ± 1.7
CIS-RRMS	64 ± 6	18 ± 5	1.6 ± 1	8 ± 3.4	1.8 ± 1.5	2 ± 1.4
SPMS	63 ± 11	24 ± 9	5.5 ± 7	1.4 ± 1.1	0.5 ± 0.5	2.5 ± 2.1
Lues	54 ± 17	19 ± 4	1.3 ± 1	17 ± 13	0.5 ± 0.8	2.3 ± 1.2
LNB	50 ± 9	19 ± 7	1.5 ± 2	20 ± 6.2	0.4 ± 0.7	2.3 ± 1.3
Bacterial meningitis	52 ± 15	22 ± 8	1.8 ± 2	4.4 ± 5.3	0.6 ± 0.8	9.2 ± 8.4
Viral meningitis	58 ± 15	20 ± 11	3.2 ± 3	3 ± 3.9	0.4 ± 0.7	6.1 ± 2.7

**Table 3:** Significant changes of CSF immune cell subsets (absolute numbers and percentage distributions) and CSF cytokine concentrations are shown comparing neuro-inflammatory diseases with non-inflammatory neurological diseases (NIND)

Disease	Elevated CSF immune cell subtypes (absolute numbers)	Elevated CSF immune cell subsets (percentage)	Elevated CSF cytokine concentration
CIS/RRMS	CD4 T cells ↑** CD8 T cells ↑*** B cells ↑*** Plasmablasts ↑*** NK cells ↑*	B cells ↑*** Plasmablasts ↑***	CCL22 ↑*, CXCL13 ↑***
SPMS			
Lues	CD4 T cells ↑* CD8 T cells ↑* B cells ↑**	B cells ↑**	CXCL11 ↑*, CXCL13 ↑*
LNB	CD4 T cells ↑** CD8 T cells ↑** B cells ↑***	CD4 T cells ↓* B cells ↑***	CCL3 ↑*, CCL7 ↑*, CCL8 ↑*, CXCL11 ↑*, CXCL13 ↑**, CXCL9 ↑**, IL2 ↑*
Bacterial meningitis	CD4 T cells ↑** CD8 T cells ↑** B cells ↑** NK cells ↑**	CD4 T cells ↓* B cells ↑*	CCL1 ↑**, CCL11 ↑*, CCL13 ↑**, CCL19 ↑*, CCL20 ↑**, CCL23 ↑*, CCL25 ↑**, CCL3 ↑**, CCL7 ↑**, CCL8 ↑**, CXCL1 ↑**, CXCL11 ↑*, CXCL13 ↑**, CXCL2 ↑**, CXCL6 ↑**, CXCL8 ↑**, CXCL9 ↑**, IFNy ↑**, IL10 ↑**, IL16 ↑**, IL2 ↑**, IL6 ↑*, TNFα ↑**
Viral meningitis	CD4 T cells ↑** CD8 T cells ↑** B cells ↑** NK cells ↑**	B cells ↑* NK cells ↑*	CCL1 ↑*, CCL19 ↑*, CCL20 ↑*, CCL22 ↑**, CCL3 ↑*, CCL7 ↑*, CCL8 ↑*, CX3CL1 ↑*, CXCL11 ↑**, CXCL12 ↑*, CXCL13 ↑**, CXCL2 ↑*, CXCL6 ↑*, CXCL9 ↑**, IFNy ↑*, IL16 ↑*

Significances after Bonferroni’s correction: *p value < 0.05; **p value < 0.01; ***p value < 0.001; arrows indicate elevated (↑) or decreased (↓) values

In order to obtain a more detailed picture, we also analyzed changes in percentage distributions for the different diseases, using NIND as a control group (Table 2). An elevated fraction of B cells was observed in patients with CIS-RRMS, Lues, LNB, and bacterial and viral meningitis, consistent with the absolute count of CSF immune cell subtypes. Plasmablasts were only significantly elevated in CIS-RRMS. NK cell percentage was significantly increased in viral meningitis. In contrast, the CD4 T cell fraction was significantly reduced in LNB and bacterial meningitis most likely due to a relative percentage increase of other populations. Interestingly, when comparing samples with Q_Albumin_ < 8 versus Q_Albumin_ ≥ 8, the immune cell percentage did not differ significantly for all subtypes except for NK cells (on average 2.5% versus 4.9% in patients with Q_Albumin_ < 8 versus Q_Albumin_ ≥ 8; Wilcoxon test, *p* < 0.003).

### Cytokine concentrations in the CSF and serum

**Fig. 1:**
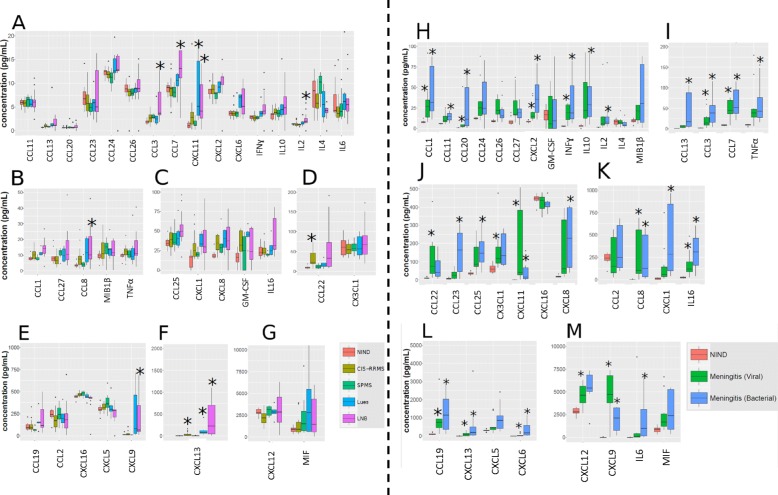
Boxplot diagrams of all CSF cytokine concentrations. For better illustration, patients are divided into a patient group with NIND, CIS/RRMS, SPMS, Lues, and LNB (**a**–**g**) and into a patient group with NIND and bacterial and viral meningitis (**h**–**m**). Diagrams are further grouped according to the ranges of cytokine concentrations with 0–20 pg/mL (**a**), 0–50 pg/mL (**b**), 0–100 pg/mL (**c**), 0–200 pg/mL (**d**), 0–1000 (**e**), 0–2000 pg/mL (**f**), 0–10,000 pg/mL (**g**), 0–100 pg/mL (**h**), 0–200 pg/mL (**i**), 0–500 pg/mL (**j**), 0–1000 pg/mL (**k**), 0–5000 pg/mL (**l**), and 0–10,000 pg/mL (**m**). Significant differences in cytokine concentrations between neuro-inflammatory diseases and patients with NIND are marked with asterisks (*) (for detailed significance levels, please refer to Table 2). NIND, non-inflammatory neurological diseases; CIS, clinically isolated syndrome; RRMS, relapsing-remitting multiple sclerosis; SPMS, secondary progressive multiple sclerosis; LNB, Lyme neuro-borreliosis

CSF concentrations of all cytokines were analyzed by comparing values from different neuro-inflammatory diseases with NIND. Multiple cytokines (26/36) showed significantly elevated CSF concentrations under conditions of neuro-inflammation (Fig. 1, Table 2). CSF concentrations of CXCL13 were significantly elevated in patients with CIS-RRMS, Lues, LNB, and bacterial and viral meningitis, CXCL11 CSF concentrations in all patients groups except CIS/RRMS and SPMS. The chemokines CCL3, CCL7, CCL8, and CXCL9 were all significantly elevated in patients with LNB and bacterial and viral meningitis. Both, bacterial and viral meningitis additionally displayed significantly elevated concentrations of the cytokines CCL1, CCL19, CCL20, CXCL2, CXCL6, IFNγ, and IL16. A significant increase of CCL22 concentrations was observed in CIS-RRMS and viral meningitis. CX3CL1 and CXCL12 were only found elevated in viral meningitis. CCL11, CCL13, CCL23, CCL25, CXCL1, CXCL8, IL6, IL10, and TNFα were significantly elevated in bacterial meningitis only, and IL2 in bacterial meningitis and in patients with LNB. No significant changes were observed for the cytokines CCL2, CCL24, CCL26, CCL27, CXCL16, CXCL5, GM-CSF, IL4, MIF, and MIB1b (10 out of 36) (Table 3).

Patients with a disrupted blood-brain barrier (BBB) defined by albumin quotient ≥ 8 showed significantly higher cytokine levels within CSF for 29 out of 36 (81%) cytokines when compared to patients with albumin quotient < 8 (Wilcoxon test, *p* value < 0.05). CXCL16 and GM-CSF showed significantly lower concentrations (Wilcoxon test, *p* value < 0.05) whereas IL4, CCL2, CXCL5, MIF, and MIB1β values did not show significant differences. We found no evidence that gender nor freezing time had an impact on overall cytokine levels in the serum or CSF. However, MIF serum concentrations correlated with freezing time (*p* value < 0.05) in patients with LNB and age correlated with CCL27 CSF concentrations (*p* value < 0.01) in patients with CIS-RRMS.

Serum concentrations of measured cytokines are shown in Additional file 1: Figure S1; significant changes were only observed for CCL3, CXCL8, and IL6 with significantly lower concentrations in patients with CIS-RRMS when compared to patients with NIND.

### Correlation analyses between CSF cytokine concentrations and CSF parameters

We performed correlations among CSF cytokine concentrations themselves, CSF, and serum concentrations and correlations between CSF cytokine concentrations and CSF parameters including CSF immune cell distributions.

Within the CSF compartment, we observed significant correlations among 29 out of 36 cytokines (> 24 correlations for each CSF cytokine, Additional file 2: Figure S2). Correlations were only restricted for CCL2, GM-CSF, CXCL13, CXCL16, MIB1β, MIF, and IL4 (≤ 24 correlations for each CSF cytokine, on average 13 correlations) indicating that these cytokines might be regulated more independently. CXCL16 mainly showed negative correlations with other cytokines suggesting a downregulation during neuro-inflammation. We also examined cytokine correlations between CSF and serum concentrations in order to discriminate to what extent a passive transfer from the periphery into the CSF, or vice versa, might occur. Only 3/36 cytokines showed a significant correlation between CSF and serum values, namely CCL23, CCL27, and IL6 (Additional file 3: Figure S3).

**Fig. 2:**
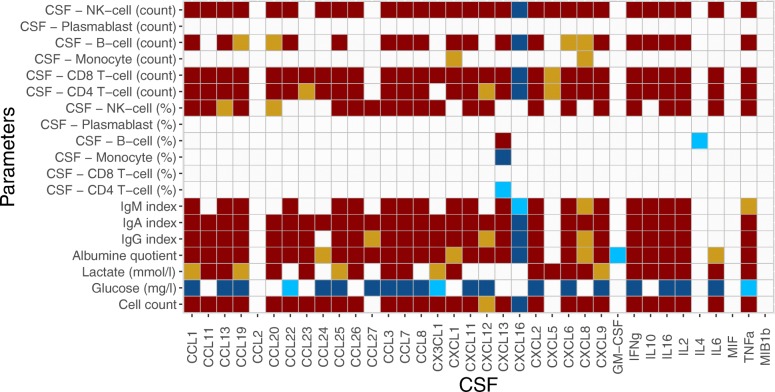
Heatmap representing significant correlations between CSF cytokine concentrations and CSF parameters including cell count, glucose, lactate, Q_Albumin_, Ig indices, percentage of immune cell distribution, and absolute immune cell numbers in the CSF. Positive correlations are given in red, and negative correlations in blue. Only correlations with *p* value < 0.05 after Bonferroni’s correction are displayed

Concerning standard CSF parameters (Fig. 2), CSF cell count significantly correlated with 29 out of 36 cytokines (all except CCL2, CCL27, CXCL5, GM-CSF, IL-4, MIF, and MIB1β) and Q_Albumin_ significantly correlated with 31 out of 36 cytokines (all except CCL2, CXCL5, IL4, MIF, and MIB1β). Interestingly, CXCL16 and GM-CSF showed a negative correlation with Q_Albumin_. Multiple negative correlations were observed between CSF cytokines and glucose levels (21/36), and positive correlations between CSF cytokines and lactate levels (23/36). In regard to an intrathecal immunoglobulin synthesis, IgA index significantly correlated with 29 out of 36 cytokines (all except CCL2, CXCL5, GM-CSF, IL4, IL6, MIF, and MIB1β), and IgG with 28 out of 36 cytokines (all except CCL2, CCL24, CXCL5, GM-CSF, IL4, IL6, MIF, and MIB1β); IgM index showed a significant correlation with 22 out of 36 cytokines (all except CCL11, CCL2, CCL20, CCL23, CCL24, CCL27, CX3CL1, CXCL12, CXCL5, GM-CSF, IL4, IL6, MIF, and MIB1β) (Fig. 2).

Correlations between total numbers of CSF immune cell subsets and CSF cytokines were mainly driven by the CSF absolute white blood cell count (Fig. 2). To evaluate distinct effects between cytokines and immune cells, we analyzed the percentage distribution of immune cell subsets. CD4 T cell and monocyte percentage showed a significantly negative correlation with CXCL13. The fraction of B cells showed a significant positive correlation with CXCL13 and negative correlation with IL4. NK cells showed a significant positive correlation with multiple cytokines, namely CCL1, CCL11, CCL13, CCL19, CCL20, CCL25, CCL26, CCL27, CCL3, CCL7, CCL8, CX3CL1, CXCL11, CXCL12, CXCL2, CXCL6, CXCL9, IFNγ, IL16, IL2, IL6, and TNFα (Fig. 2).

### Correlation analyses between CSF cytokine concentrations and CSF parameters with respect to albumin quotient

**Fig. 3:**
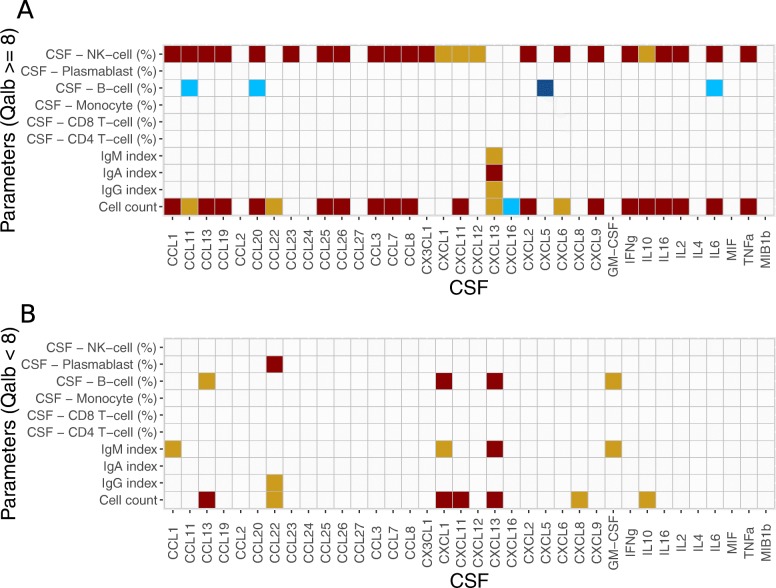
Correlation subanalyses of CSF cytokines and a subset of CSF parameters including CSF cell count, Q_Albumin_, Ig indices, and percentage distribution of immune cell subsets in patients with **a** Q_Albumin_ ≥ 8 and **b** Q_Albumin_ < 8. Positive correlations are given in red, and negative correlations in blue. Only correlations with *p* value < 0.05 after Bonferroni’s correction are displayed

Since most of the cytokines correlated with Q_Albumin_, we divided patients with Q_Albumin_ ≥ 8 (*n* = 39, disrupted blood-brain barrier) and Q_Albumin_ < 8 (*n* = 36, intact blood-brain barrier) into two different groups for further analyses (Fig. 3).

Correlation analyses between different cytokines within the CSF compartment again showed multiple correlations in patients with a Q_Albumin_ ≥ 8 (on average with 29/36 cytokines, Additional file 4: Figure S4). Again, CCL2, GM-CSF, CXCL13, CXCL16, MIB1β, MIF, and IL4 and in addition CCL22, CCL24, CCL27, and CXCL5 showed limited correlations (on average with 4/36 cytokines). In general, correlations between CSF cytokines in patients with Q_Albumin_ < 8 were limited (average number of correlations 6/36, Additional file 5: Figure S5). In line with results from all samples, only CCL27 values correlated between CSF and serum in patients with a Q_Albumin_ ≥ 8; in samples with a Q_Albumin_ < 8, CCL23 and CCL27 values correlated between CSF and serum indicating that a disrupted blood-brain barrier does not lead to a primarily passive exchange of cytokines between both compartments.

Regarding CSF cell count, patients with a Q_Albumin_ ≥ 8 still showed significant correlations with multiple cytokines including CCL1, CCL11, CCL13, CCL19, CCL20, CCL22, CCL25, CCL26, CCL3, CCL7, CCL8, CXCL11, CXCL13, CXCL16, CXCL2, CXCL6, CXCL9, IFNγ, IL10, IL16, IL2, IL6, and TNFα. Patients with a Q_Albumin_ < 8 only showed a correlation between cytokines and CSF cell count for a limited number of cytokines including CCL13, CCL22, CXCL1, CXCL11, CXCL13, CXCL8, and IL10. The IgA, IgG, and IgM indices only correlated with CXCL13 in patients with a Q_Albumin_ ≥ 8. Patients with a Q_Albumin_ < 8 showed a correlation between IgG index and CCL22 and between IgM index and the cytokines CCL1, CXCL1, CXCL13, and GM-CSF.

When looking at the correlations between the percentage of different immune cell subtypes and cytokine concentrations, differential results could be observed. Similar to the analysis with all patients, samples with a Q_Albumin_ ≥ 8 showed a positive correlation between NK cell percentage and CCL1, CCL11, CCL13, CCL19, CCL20, CCL23, CCL25, CCL26, CCL3, CCL7, CCL8, CX3CL1, CXCL1, CXCL11, CXCL12, CXCL2, CXCL6, CXCL9, IFNγ, IL10, IL16, IL2, IL6, and TNF-α. B cell percentage negatively correlated with CCL11, CCL20, CXCL5, and IL6. Patients with a Q_Albumin_ < 8 did not show any correlations between cytokines and NK cells. For the B cell fraction, samples with a Q_Albumin_ ≥ 8 showed results with negative correlations for CCL11, CCL20, CXCL5, and IL6. However, samples with Q_Albumin_ < 8 displayed a positive correlation between B cells and CCL13, CXCL1, CXCL13, and GM-CSF, and a correlation between plasmablasts and CCL22.

## Discussion

In order to further understand neuro-inflammatory processes in regard to CSF cytokine profiles and immune cell subtypes, we studied CSF and serum concentrations of 36 cytokines in combination with standard CSF parameters and CSF distributions patterns of CD4 and CD8 T cells, B cells, plasmablasts, monocytes, and NK cells in 75 neurological patients. We observed an upregulation of multiple cytokines during neuro-inflammation. Correlation analyses revealed that B cell activation together with an upregulation of CXCL13 occurs under conditions of an intact blood-brain barrier (BBB). Under conditions of a disrupted BBB, NK cells significantly increased and seemed to have a major contribution to inflammatory processes, which was reflected by a strong correlation with multiple pro-inflammatory cytokines.

Analyzing all samples together, the majority of measured cytokines (26/36) showed elevated CSF values under certain inflammatory conditions. Most of these CSF cytokines correlated with each other (31/36) and with routine CSF parameters such as CSF cell count (29/36) and Q_Albumin_ (31/36), indicating that an upregulation of CSF immune cells and disruption of the blood-brain barrier are associated with numerous pro-inflammatory cytokines. However, the cytokines CCL2, CXCL5, IL4, MIF, and MIB1β did not show a significant elevation or correlation with CSF cell count and Q_Albumin_, so that these cytokines are most likely not involved in inflammatory CSF processes. In patients with a disrupted blood-brain barrier, only CCL27 concentrations correlated between CSF and serum. Thus, a passive exchange of cytokines across the BBB does not seem to be the major source of CSF cytokines in neuro-inflammation. Instead, a local production and parallel upregulation of cytokines seem more plausible. These results suggest that a cascade of intrathecal cytokine production and immune cell recruitment in combination with a disruption of the BBB occurs during neuro-inflammation. The chronology of cytokine expression during these neuro-inflammatory processes cannot be determined by correlation analyses, but certain hints can be drawn from further detailed analyses.

Regarding the different immune cell subtypes, NK cells turned out to be a prominent cell population that might support neuro-inflammatory processes especially under conditions of a disrupted BBB. Both NK cell percentage and cytokine concentrations in the CSF were significantly higher in patients with a disrupted BBB. Consequently, NK cells showed a strong correlation with multiple CSF cytokines in those patients whereas patients with an intact BBB did not show any correlations at all. Pro-inflammatory cytokines such as TNF-α and IL6 have been shown to be involved in BBB breakdown [8, 24, 33] which was also reflected in our analyses with correlations between TNF-α and IL6, and NK cells as wells as Q_Albumin_. Possible chemotactic factors for intrathecal NK cell recruitment are CXCL8, CCL3, and CX3CL1 [30]. Indeed, CCL3 and CX3CL1 both correlated with NK cells in our data. During the interaction with other immune cells, NK cells release high amounts of IFNγ and TNFα in addition CCL3, CCL4, and CCL5 [2, 11, 30]. We could confirm a positive correlation between IFNγ, TNFα, CCL3, and NK cells (CCL4 and CCL5 not included in our analysis) which could point towards a possible intrathecal production of these cytokines by NK cells. Taken together, the correlation with multiple CSF cytokines and NK cells might reflect several actions including NK cell recruitment and effects on the BBB, production of cytokines through NK cells, and interaction with other immune cell types.

Another strong association could be found between CSF B cells and CXCL13 values which is in line with previous results [20]. In contrast to NK cells, CXCL13 correlated with B cells under conditions of an intact BBB. Thus, the upregulation of CSF B cells might occur independently of a disrupted BBB and an overall inflammatory response, which is in line with results from specific diseases such as multiple sclerosis [28]. Moreover, CSF B cells show a consistent negative correlation with CSF monocytes. CXCL13 values also show a negative correlation with CSF monocytes [20], which was confirmed in the present analysis in patients with neuro-inflammation. In addition, B cells correlated with GM-CSF (granulocyte-macrophage colony-stimulating factor) under conditions of an intact BBB, which is known to stimulate the differentiation/maturation of monocytes into macrophages [13]. In conclusion, B cells could possibly produce GM-CSF within the CSF compartment leading to a differentiation of CSF monocytes into macrophages and migration of macrophages into CNS tissues. Vice versa, CXCL13 has been reported to be produced by differentiated macrophages [5], which could influence B cell recruitment into the CSF. No consistent positive correlations were found between CD4 and CD8 T cells and cytokines which could possibly require a more detailed characterization of T cell subtypes.

When analyzing our data according to disease-specific changes, the following conclusions can be drawn for certain cytokines. The CSF milieu in multiple sclerosis has been intensively studied [19], and we could confirm a significant upregulation of CCL22 and CXCL13 in active MS patients [17, 39]. We also observed significantly different CXCL13 values between patients with CIS-RRMS and SPMS which again highlights the role of CXCL13 as a marker for active MS. In line with the literature, our analyses also confirmed elevated cytokine levels for CXCL13 and CCL3 in patients with LNB [37]. In bacterial meningitis, the majority (23/36) of examined cytokines displayed elevated values with CCL7, TNF-α, CXCL1, and IFNγ showing consistent results with the literature ([1, 35]; Pinto [7, 32, 36, 44]). Also, in viral meningitis, multiple cytokines (16/36) were elevated and we could confirm previously reported results for CXCL9, CXCL11, and IFNγ [14, 16]. When comparing cytokine levels between viral and bacterial meningitis, CCL23 and CXCL6 showed the biggest differences between both diseases but differences did not remain significant when applying Bonferroni’s correction. Further testing on larger patient collectives will be necessary for further evaluation.

The following limitations of our study have to be discussed. First, we limited the total number of patients to 75, so that they fitted on 1 plate for CSF and 1 plate for serum samples, along with the standard curve, in order to avoid interplate variation for the multiplex assays that was observed in preliminary experiments with the same samples on different plates. Second, correlations between different CSF parameters do not prove causality, so that direct conclusions on functional aspects remain speculative. Thus, multiple correlations in our studies have to be interpreted at a descriptive level. Third, an elevated Q_Albumin_ was used as a clinically pragmatic way to define a disrupted BBB but might not accurately reflect the actual barrier for cytokines, which are small molecules and might show transfer kinetics different from albumin. Fourth, we used Bonferroni’s correction for all analyses to reduce the number of false positives at the cost of having more false negatives. This strict correction for multiple testing can possibly explain differences to previous studies that measured smaller subsets of cytokines.

## Conclusion

We could show that B cell activation with an upregulation of CXCL13 can occur under conditions of a grossly intact blood-brain barrier. After break down of this barrier, NK cells significantly increased and seemed to have a major contribution to inflammatory processes, which was reflected by a strong correlation with multiple cytokines. From a clinical point of view, CXCL13 was again confirmed as a reliable marker for CSF B cell recruitment and might be used as a clinical marker to predict disease activity in MS, confirming the diagnosis of LNB and CNS lymphoma [12]. However, cytokine profiles need further evaluation in bigger and more homogenous disease groups. Future studies are necessary to address the exact kinetics of these cytokines and their relation to CSF immune cell subtypes during neuro-inflammation in the context of specific disease phenotypes.

## Supplementary information

**Additional file 1: Figure S1.** Boxplot diagrams of all serum cytokine concentrations including all patient groups (NIND, CIS/RRMS, SPMS, Lues and bacterial and viral meningitis). Diagrams are further grouped according to ranges of concentrations with 0–25 pg/mL (A), 0–100 pg/mL (B), 0–200 pg/mL (C), 0–500 pg/mL (D), 0–1000 (E) and 0–5000 pg/mL (F).

**Additional file 2: Figure S2.** Heatmap representing correlations between different CSF cytokines among each other. Positive correlations are given in red, negative correlations in blue. Only correlations with *p*-value < 0.05 after Bonferroni correction are displayed.

**Additional file 3: Figure S3.** Heatmap representing correlations between CSF and serum cytokine concentrations. Positive correlations are given in red, negative correlations in blue. Only correlations with p-value < 0.05 after Bonferroni correction are displayed.

**Additional file 4: Figure S4.** Heatmap representing correlations between different CSF cytokines among each other for patients with Q_albumin_ ≥ 8. Positive correlations are given in red, negative correlations in blue.

**Additional file 5: Figure S5.** Heatmap representing correlations between different CSF cytokines among each other for patients with Q_albumin_ < 8. Positive correlations are given in red, negative correlations in blue. Only correlations with p-value < 0.05 after Bonferroni correction are displayed.

**Additional file 6: Table S1.** Measured standard curves in CSF and serum and sensitivity values provided by the manufacturer. Measured standard curves above had a wide linear range and showed very close values to the ranges provided for serum values by the manufacturer. N.A. not provided by manufacturer.

**Additional file 7: Table S2.** Routine CSF parameters including CSF cell count, glucose, lactate, Q_Albumin_ and Ig indices are shown for different diseases. Average values with ± standard deviations are displayed.

## Data Availability

The R script is available at https://figshare.com/articles/2019_11_04_Lepennetier_et_all_2019_Journal_Neuroinflammation/10247471 and the raw data at https://figshare.com/articles/raw_data/10247474

## Abbreviations

BBB: Blood-brain barrier
CIS: Clinically isolated syndrome
CSF: Cerebrospinal fluid
FACS: Fluorescence-activated cell sorting
LNB: Lyme neuro-borreliosis
NK cells: Natural killer cells
RRMS: Relapsing-remitting multiple sclerosis
SPMS: Secondary progressive multiple sclerosis
