# Supplementary figures and images for: Cytokine and immune cell profiling in the cerebrospinal fluid of patients with neuro-inflammatory diseases
Source: J Neuroinflammation. 2019 Nov 14;16:219. doi: 10.1186/s12974-019-1601-6 (PMC6857241; doi:10.1186/s12974-019-1601-6)

## Slide 1
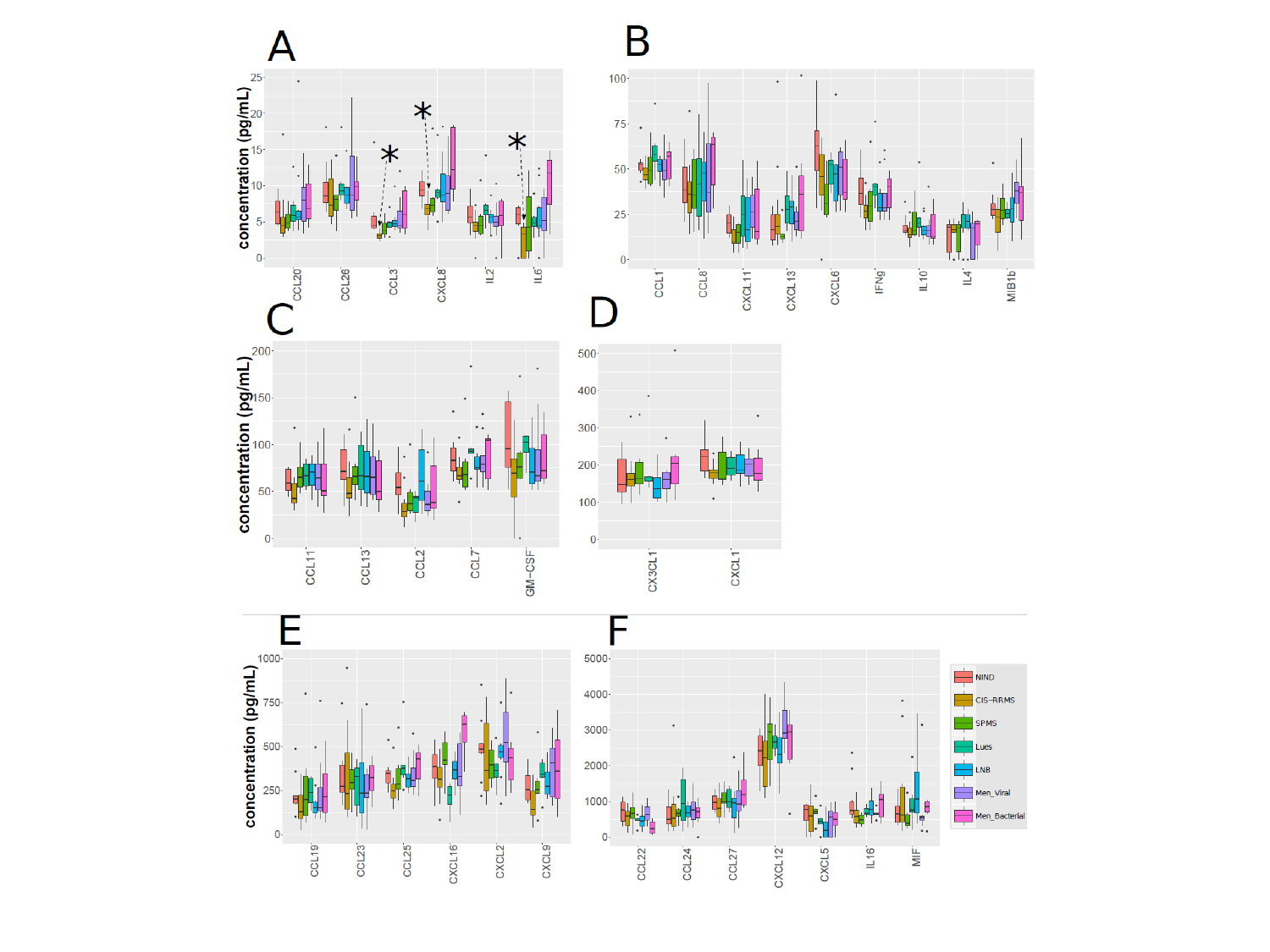

Supplement: Supplementary file 1 — Additional file 1: Figure S1. Boxplot diagrams of all serum cytokine concentrations including all patient groups (NIND, CIS/RRMS, SPMS, Lues and bacterial and viral meningitis). Diagrams are further grouped according to ranges of concentrations with 0–25 pg/mL (A), 0–100 pg/mL (B), 0–200 pg/mL (C), 0–500 pg/mL (D), 0–1000 (E) and 0–5000 pg/mL (F). [file 12974_2019_1601_MOESM1_ESM.pptx]

CSF

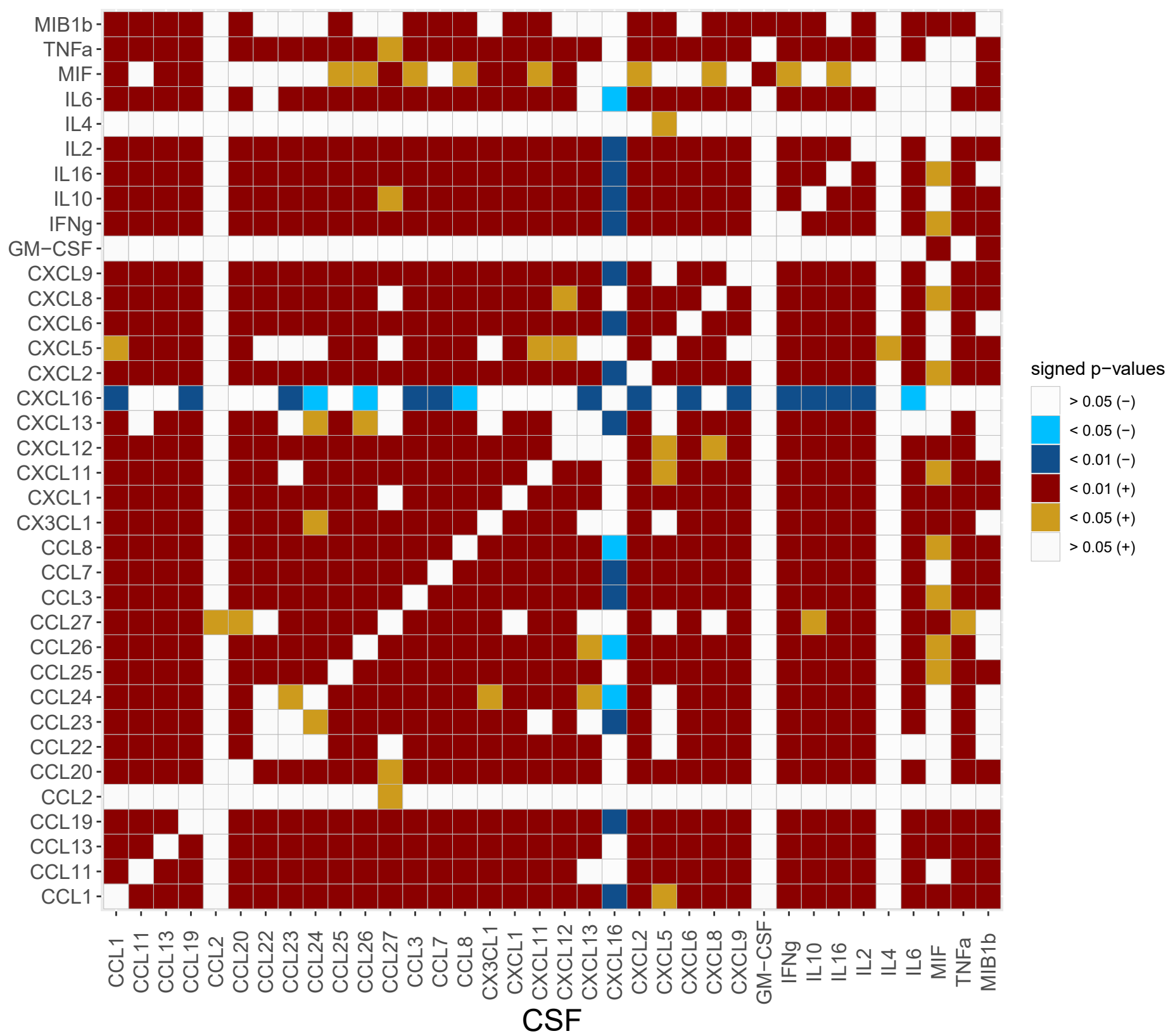

Supplement: Supplementary file 2 — Additional file 2: Figure S2. Heatmap representing correlations between different CSF cytokines among each other. Positive correlations are given in red, negative correlations in blue. Only correlations with p-value < 0.05 after Bonferroni correction are displayed. [file 12974_2019_1601_MOESM2_ESM.pdf]

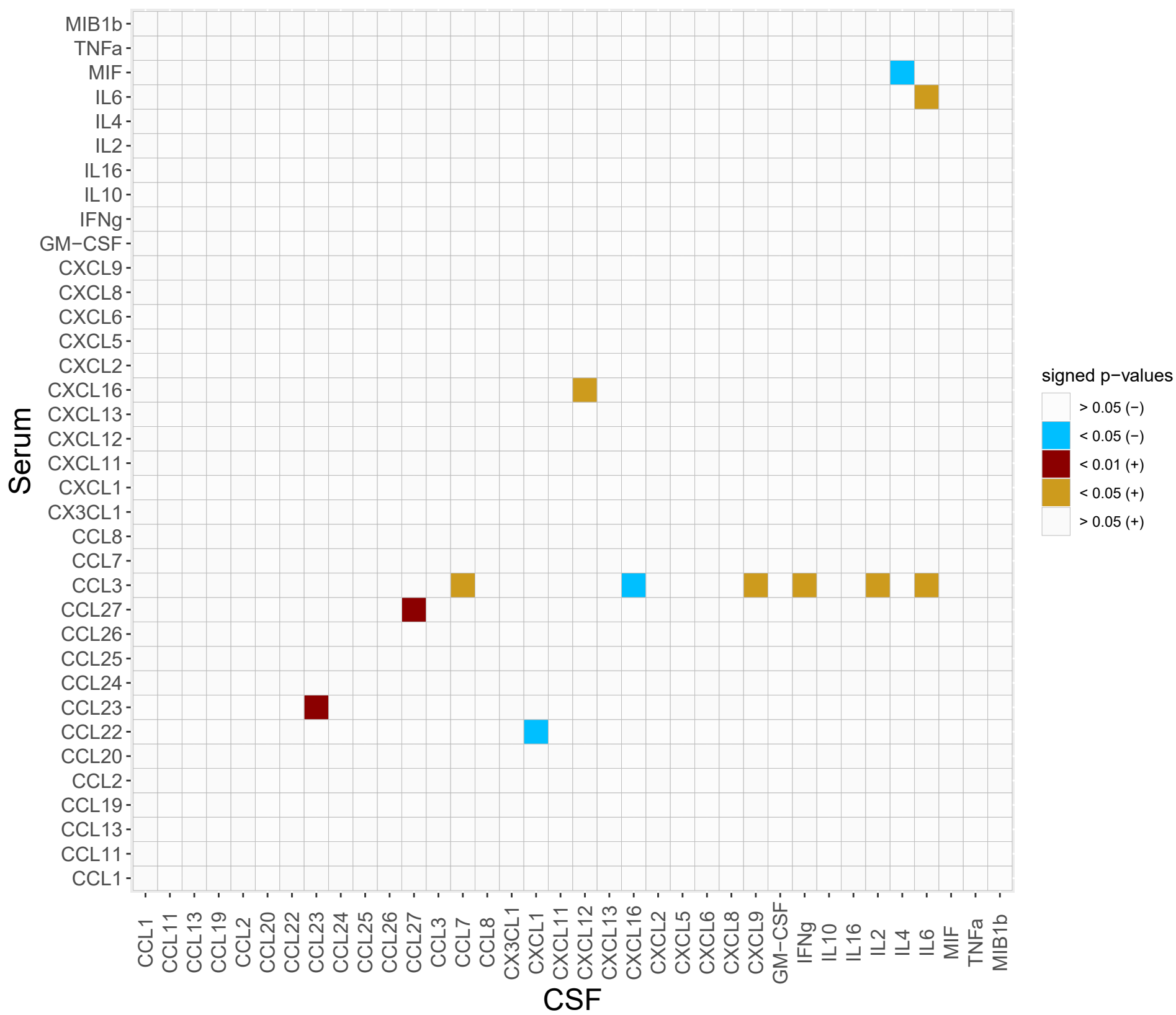

Supplement: Supplementary file 3 — Additional file 3: Figure S3. Heatmap representing correlations between CSF and serum cytokine concentrations. Positive correlations are given in red, negative correlations in blue. Only correlations with p-value < 0.05 after Bonferroni correction are displayed. [file 12974_2019_1601_MOESM3_ESM.pdf]

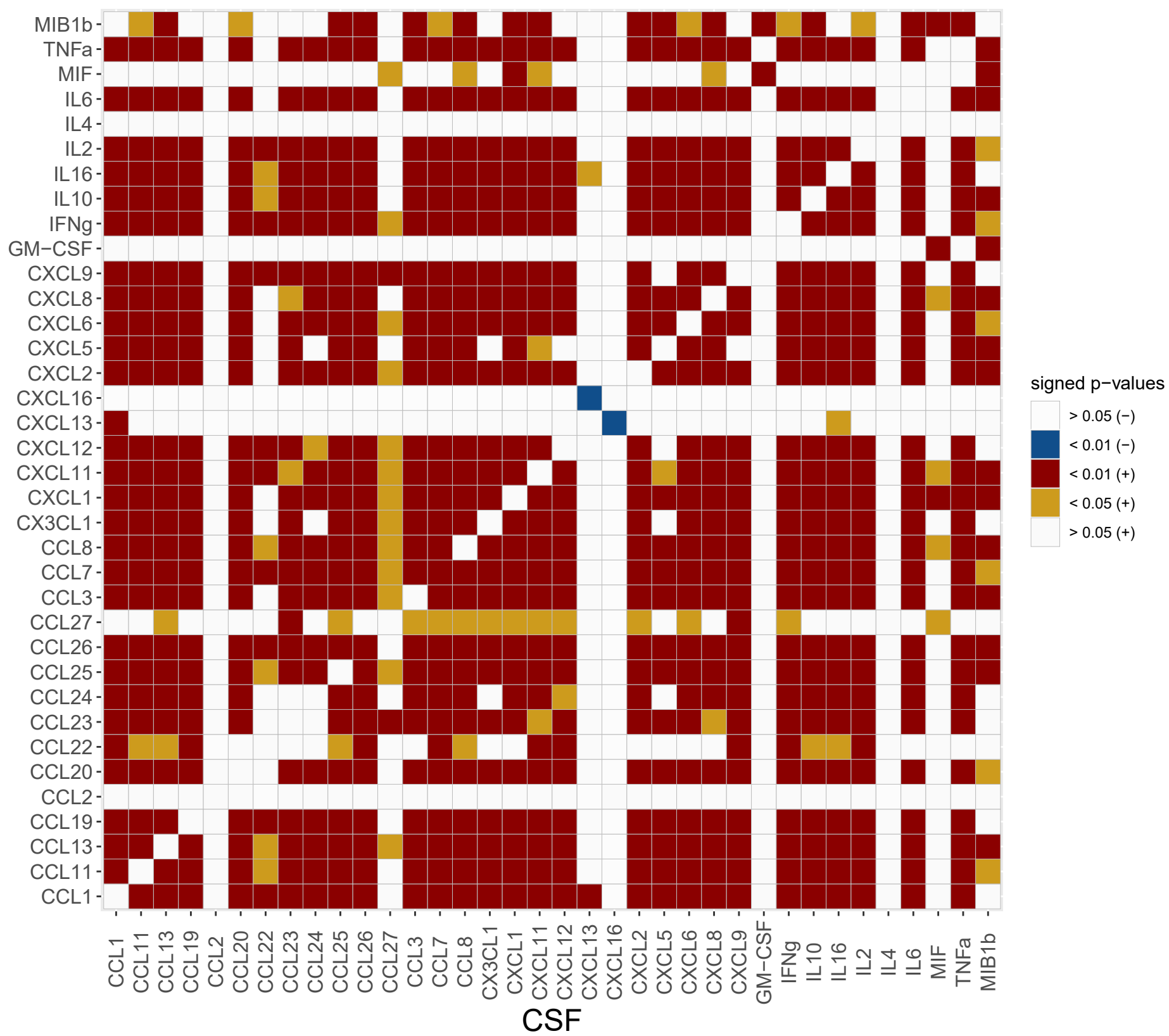

Supplement: Supplementary file 4 — Additional file 4: Figure S4. Heatmap representing correlations between different CSF cytokines among each other for patients with Qalbumin ≥ 8. Positive correlations are given in red, negative correlations in blue. [file 12974_2019_1601_MOESM4_ESM.pdf]

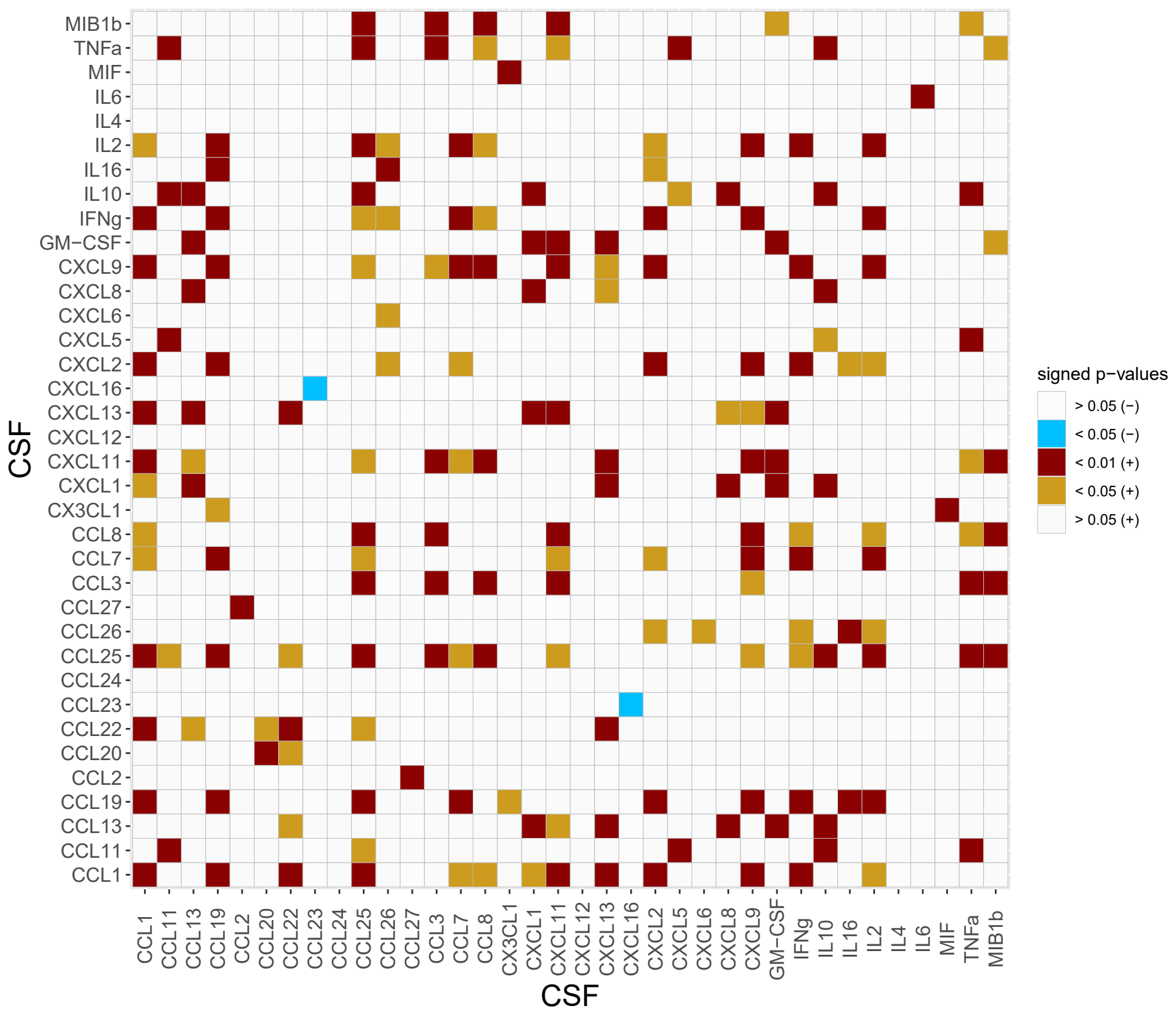

Supplement: Supplementary file 5 — Additional file 5: Figure S5. Heatmap representing correlations between different CSF cytokines among each other for patients with Qalbumin < 8. Positive correlations are given in red, negative correlations in blue. Only correlations with p-value < 0.05 after Bonferroni correction are displayed. [file 12974_2019_1601_MOESM5_ESM.pdf]
