# Supplementary material for: Cytokine and immune cell profiling in the cerebrospinal fluid of patients with neuro-inflammatory diseases
Source: J Neuroinflammation. 2019 Nov 14;16:219. doi: 10.1186/s12974-019-1601-6 (PMC6857241; doi:10.1186/s12974-019-1601-6)
Supplement: Supplementary file 6 — Additional file 6: Table S1. Measured standard curves in CSF and serum and sensitivity values provided by the manufacturer. Measured standard curves above had a wide linear range and showed very close values to the ranges provided for serum values by the manufacturer. N.A. not provided by manufacturer. [file 12974_2019_1601_MOESM6_ESM.docx]

***Supplementary Table 1****: Measured standard curves in CSF and serum and sensitivity values provided by the manufacturer.*

|  | Standard curve CSF | | Standard curve Serum | | Sensitivity |  |
| --- | --- | --- | --- | --- | --- | --- |
| Cytokine | | Lower limit | Upper limit | Lower limit | Upper limit | in pg/ml provided by company |
| CCL21 | | 37.09 | 28696.57 | 46.62 | 2552.6 | 12.0 |
| CXCL13 | | 0.42 | 5852.77 | 0.34 | 1410.24 | 0.1 |
| CCL27 | | 1.16 | 16873.53 | 1.2 | 4826.8 | 0.3 |
| CXCL5 | | 8.89 | 194108.67 | 42.92 | 54335.1 | 5.7 |
| CCL11 | | 0.91 | 200.74 | 0.94 | 3661.33 | 0.7 |
| CCL24 | | 1.59 | 1529.18 | 3.006 | 11870.98 | 3.2 |
| CCL26 | | 6.18 | 26707.12 | 0.49 | 2071.76 | 0.5 |
| CX3CL1 | | 2.95 | 10367.3 | 4.25 | 19490.1 | 0.9 |
| CXCL6 | | 0.6 | 13307.72 | 3.72 | 4149.38 | 0.6 |
| GM-CSF | | 8.64 | 34473.21 | 8.63 | 9225 | 1.0 |
| CXCL1 | | 13.75 | 11804.3 | 11.42 | 12595.36 | 4.2 |
| CXCL2 | | 4.35 | 20593.37 | 4.31 | 4821.3 | 2.7 |
| CCL1 | | 2.15 | 7663.01 | 2.25 | 7782.36 | 1.6 |
| IFNɣ | | 1.69 | 7184.4 | 1.99 | 8957.17 | 0.4 |
| IL1β | | 0.55 | 7235.82 | 0.73 | 3071.99 | 0.1 |
| IL2 | | 0.81 | 13264.57 | 0.67 | 2879.63 | 0.1 |
| IL4 | | 1.14 | 1122.31 | 3.67 | 4134.32 | 1.0 |
| IL6 | | 0.7 | 11283.13 | 0.69 | 2833.16 | 0.1 |
| CXCL8 | | 7.41 | 8173.05 | 0.43 | 1863.85 | 0.04 |
| IL10 | | 1.15 | 1186.94 | 1 | 4951.16 | 0.9 |
| IL16 | | 2.25 | 34571.63 | 9.48 | 37297.1 | 0.8 |
| CXCL10 | | 3.98 | 1243.96 | 1.03 | 4946.94 | 1.1 |
| CXCL11 | | 0.23 | 2894.23 | 0.13 | 543.15 | 0.05 |
| CCL2 | | 0.28 | 3967.09 | 0.21 | 919.64 | 0.1 |
| CCL8 | | 0.28 | 3951.31 | 0.26 | 1103.01 | 0.04 |
| CCL7 | | 2.11 | 7714.62 | 1.32 | 5975.84 | 1.3 |
| CCL13 | | 0.21 | 3278.83 | 0.17 | 726.54 | 0.1 |
| CCL22 | | 1.35 | 16899.13 | 1.1 | 4705.23 | 0.5 |
| MIF | | 25.2 | 121619.2 | 20.13 | 87799.56 | 15.4 |
| CXCL9 | | 4.74 | 23142.26 | 5.66 | 23419.4 | 1.1 |
| CCL3 | | 0.35 | 1402.75 | 0.28 | 1078.25 | 0.3 |
| CCL15 | | 1.23 | 14129.48 | 1.47 | 6171.81 | 0.2 |
| CCL20 | | 0.46 | 6807.15 | 0.57 | 2316.03 | 0.1 |
| CCL19 | | 3.07 | 46962.74 | 2.28 | 9424.74 | 1.1 |
| CCL23 | | 1.05 | 14888.85 | 0.86 | 1035.87 | 0.5 |
| CXCL16 | | 0.48 | 5763.82 | 0.32 | 1343.26 | 0.1 |
| CXCL12 | | 9.97 | 126197.32 | 9.16 | 42559.73 | 10.3 |
| CCL17 | | 5.9 | 5587.58 | 1.52 | 87.87 | 1.1 |
| CCL25 | | 9.48 | 97101.21 | 6.33 | 25645.18 | 4.9 |
| TNFα | | 0.81 | 12774.55 | 0.81 | 3354.19 | 0.2 |
| MIB1β | | 0.33 | 4038.41 | 1.07 | 4374.98 | N.A |

Measured standard curves above had a wide linear range and showed very close values to the ranges provided for serum values by the manufacturer. N.A. not provided by manufacturer.
