# Supplementary material for: Cytokine and immune cell profiling in the cerebrospinal fluid of patients with neuro-inflammatory diseases
Source: J Neuroinflammation. 2019 Nov 14;16:219. doi: 10.1186/s12974-019-1601-6 (PMC6857241; doi:10.1186/s12974-019-1601-6)
Supplement: Supplementary file 7 — Additional file 7: Table S2. Routine CSF parameters including CSF cell count, glucose, lactate, QAlbumin and Ig indices are shown for different diseases. Average values with ± standard deviations are displayed. [file 12974_2019_1601_MOESM7_ESM.docx]

**Supplementary Table 2**: Routine CSF parameters including CSF cell count, glucose, lactate, Q_Albumin_ and Ig indices are shown for different diseases. Average values with ± standard deviations are displayed.

| **Clinical syndrome** | **CSF cell count (cell/μL)** | **Glucose (mg/L)** | **Lactate (mmol/L)** | **Qalb** | **Qalb >= 8 / total** | **IgG index** | **IgA index** | **IgM index** |
| --- | --- | --- | --- | --- | --- | --- | --- | --- |
| **NIND** | 1.3^±1.4^ | 67^±7^ | 1.7^±0.3^ | 5.1^±1.7^ | 0/10 | 0.46^±0.09^ | 0.23^±0.07^ | 0.03^±0.04^ |
| **CIS-RRMS** | 12^±12^ | 69^±18^ | 1.9^±0.4^ | 6.8^±3.2^ | 5/18 | 0.89^±0.51^ | 0.30^±0.06^ | 0.13^±0.10^ |
| **SPMS** | 2^±1.2^ | 70^±22^ | 1.9^±0.5^ | 8.5^±4.0^ | 4/8 | 0.65^±0.17^ | 0.26^±0.05^ | 0.08^±0.10^ |
| **Lues** | 21^±25^ | 63^±8^ | 1.6^±0.3^ | 5.9^±1.9^ | 1/6 | 0.68^±0.32^ | 0.28^±0.10^ | 0.30^±0.22^ |
| **LNB** | 62^±83^ | 58^±9^ | 1.8^±0.4^ | 15.9^±11.3^ | 11/13 | 0.75^±0.25^ | 0.79^±.0.94^ | 0.99^±1.04^ |
| **Bacterial meningitis** | 453^±1045^ | 49^±30^ | 4.4^±2.4^ | 32^±19.1^ | 10/10 | 0.62^±0.15^ | 0.40^±0.19^ | 0.22^±0.16^ |
| **Viral meningitis** | 240^±216^ | 57^±8^ | 2.8^±1^ | 18.2^±11.2^ | 8/10 | 0.51^±0.09^ | 0.34^±0.08^ | 0.12^±0.04^ |
